# Outcome predictors of intra-articular glucocorticoid treatment for knee synovitis in patients with rheumatoid arthritis – a prospective cohort study

**DOI:** 10.1186/ar4586

**Published:** 2014-06-20

**Authors:** Tomas Weitoft, Johan Rönnelid, Ann Knight, Jörgen Lysholm, Tore Saxne, Anders Larsson

**Affiliations:** 1 Section of Rheumatology, Centre for Research and Development, Uppsala University/County Council of Gävleborg, Gävle 801 87, Sweden; 2 Department of Immunology, Genetics and Pathology, Uppsala University, Uppsala, Sweden; 3 Section of Rheumatology, Department of Medical Sciences, Uppsala University, Uppsala, Sweden; 4 Clinic of Rheumatology, Falu Hospital, Falun, Sweden; 5 Section of Rheumatology, Department of Clinical Sciences, Lund University, Paradisgatan 2, Lund, Sweden; 6 Section of Clinical Chemistry, Department of Medical Sciences, Uppsala University, Uppsala, Sweden

## Abstract

**Introduction:**

Intra-articular glucocorticoid treatment (IAGC) is widely used for symptom relief in arthritis. However, knowledge of factors predicting treatment outcome is limited. The aim of the present study was to identify response predictors of IAGC for knee synovitis in patients with rheumatoid arthritis (RA).

**Methods:**

In this study 121 RA patients with synovitis of the knee were treated with intra-articular injections of 20 mg triamcinolone hexacetonide. They were followed for six months and the rate of clinical relapse was studied. Non-responders (relapse within 6 months) and responders were compared regarding patient characteristics and knee joint damage as determined by the Larsen-Dale index. In addition, matched samples of serum and synovial fluid were analysed for factors reflecting the inflammatory process (C-reactive protein, interleukin 6, tumour necrosis factor alpha, vascular endothelial growth factor), joint tissue turnover (cartilage oligomeric matrix protein, metalloproteinase 3), and autoimmunity (antinuclear antibodies, antibodies against citrullinated peptides, rheumatoid factor).

**Results:**

During the observation period, 48 knees relapsed (40%). Non-responders had more radiographic joint damage than responders (*P* = 0.002) and the pre-treatment vascular endothelial growth factor (VEGF) level in synovial fluid was significantly higher in non-responders (*P* = 0.002).

**Conclusions:**

Joint destruction is associated with poor outcome of IAGC for knee synovitis in RA. In addition, higher levels of VEGF in synovial fluid are found in non-responders, suggesting that locally produced VEGF is a biomarker for recurrence of synovial hyperplasia and the risk for arthritis relapse.

## Introduction

Intra-articular glucocorticoid treatment (IAGC) has been used in the treatment of arthritis disorders for decades. Glucocorticoid injection therapy is still frequently used, as signs and symptoms of synovitis can be rapidly and effectively controlled. However, despite long experience there are few investigations dealing with factors influencing the treatment outcome.

The choice of steroid preparation [1], the accuracy of the injection placement [2], the synovial fluid aspiration [3] and the immobilisation of weight-bearing joints [4] have all been identified as factors with an impact on treatment outcome. Those factors can be controlled using correct injection routines in the daily clinical practice. A successful treatment result after two weeks has also been shown to predict a favourable response at six months [5].

However, several factors, such as disease activity, degree of joint destruction, the character of the local inflammatory process and the autoimmune profile, have also been considered to be important for treatment outcome. Most investigations are small and differences in study design and outcome measures preclude firm conclusions. A high proportion of polymorphonuclear cells in synovial fluid [6] and macrophages in synovial biopsies [7] have been reported to be associated with a better treatment result in adult patients with arthritis of the knee.

Recently the Danish CIMESTRA study [8] observed a significant, but weak association, not only between the outcome of IAGC and joint erosion score for wrists using magnetic resonance imaging (MRI), but also for the presence of antibodies against citrullinated peptides (anti-CCP) in serum.

The aim of the present investigation was to identify patient or disease characteristics including biochemical variables analysed in synovial fluid and blood that may influence the long-term effect of intra-articular glucocorticoid therapy for knee synovitis in RA.

The results may be clinically important as patients at risk for early arthritis relapse could benefit from an extra careful and strict injection procedure and a better follow up regimen.

## Methods

At the outpatient rheumatology departments at the hospitals in Gävle, Uppsala and Falun, Sweden, patients with rheumatoid arthritis (RA) [9] and signs and symptoms of synovitis of the knee were invited to participate in the study. Patients with an interval <3 months since the latest injection, oral corticosteroid treatment corresponding to 10 mg prednisolone daily or more, and patients in function class 4 according to Steinbrocker [10] were excluded.

Information on patient characteristics (age, sex, body weight, disease duration, smoking habits and concomitant treatments) was collected and serum samples were drawn. The level of disability was evaluated using the Swedish version of the Health Assessment Questionnaire (HAQ) [11]. The number of tender and swollen joints were counted and the disease activity score (DAS28) [12] was calculated. Using a lateral approach the joint was entered with a 0.7 × 40 mm needle. As much as possible of the synovial fluid was aspirated and saved for analysis. A dose of 20 mg triamcinolone hexacetonide (THA) was injected. After treatment the patients were recommended a 24-hour post-injection rest at home. Within a two-month period after injection a radiological examination of the knee was performed and the radiographs were graded on a scale from 0 to 5 (0 = normal, 5 = severe changes) according to Larsen-Dale [13] by a single independent radiologist. For patients with IAGC in both knees during the study period, only the first injection was included in the study.

The patients were told to contact the rheumatology department if symptoms from the treated knee recurred. If so, the knee was examined again and if the pain or discomfort was associated with signs of clinical synovitis (tenderness and swelling) the time to relapse was registered and the patient was defined as a non-responder. The patients had follow up visits according to the clinical routines. All patients received a phone call at the end of the six-month observation period to confirm that no unregistered relapse had occurred.

Serum and synovial samples were centrifuged for 20 minutes at 1800 g within one hour and were then stored at -70°C until analysis.

The study was approved for each participating center (Gävle, Uppsala and Falun) by The Regional Ethical Review Board in Uppsala and all patients gave their informed consent.

### Laboratory methods

All analyses were performed without knowledge of the clinical outcome. The local inflammatory process was evaluated using white blood cell count of polynuclear and mononuclear cells, metalloproteinase 3 (MMP 3), tumour necrosis factor alpha (TNF), interleukin 6 (IL6) and vascular endothelial growth factor (VEGF) in synovial fluid. The four latter markers were analysed by commercial sandwich ELISA kits (DY206, DY 513, DY210 and DY293B, R&D Systems, Minneapolis, MN, USA).

Systemic inflammation and clinical disease activity were assessed by analysing serum levels of C-reactive protein (CRP), TNF, IL6, VEGF and MMP3 and calculations of the DAS28 index. CRP (reagent: 6 K2601) was analysed on an Architect Ci8200 analyser (Abbott Laboratories, Abbott Park, IL, USA).

The activity of the destructive process was assessed using analyses of serum and synovial fluid levels of the cartilage oligomeric matrix protein (COMP) and the proteolytic enzyme MMP3. COMP was analysed using a sandwich ELISA (Anamar, Lund, Sweden). The detection limit of the assay is <0.1 U/L, and its intra-assay and interassay coefficient is <5%.

The autoimmune profile of the disease was analysed using serum levels of antinuclear antibodies (ANA; ImmunoConcept; Sacramento, CA, USA, screening titer 1:200, corresponding to 95% specificity among 100 healthy controls), anti-CCP and the immunoglobulin M (IgM) subclass of Rheumatoid Factor (RF). Anti-CCP and RF were investigated with an enzyme immuno assay using a Phadia Immunocap system, Uppsala, Sweden. For anti-CCP a level of 7 arbitrary units, and for RF a level of 5 IU/ml, were regarded as positive. When investigating 100 healthy blood donors, all individuals were anti-CCP negative whereas 4% were RF positive using these cut-offs.

### Statistical methods

The results from patients with relapse within six months (non-responders) were compared with responders using statistical analysis with the *T*-test for independent values, Mann-Whitney U–test or Chi2-test when appropriate. Correlation between variables was assessed by Spearman’s rank correlation coefficient. A multivariate logistic regression model with treatment response as the dependent variable was fitted using stepwise backwards elimination of independent variables based on likelihood ratio tests. To adjust for multiple comparisons of biomarkers, Šidák correction of the significance level was performed. Correcting of a family wise error rate of 0.05 for 17 comparisons gives an adjusted significance level equal to 0.003.

The statistical calculations were made using the computer software program IBM SPSS Statistics version 21.

## Results

A total of 121 patients fulfilled the inclusion criteria and were eligible for the study and all of them could be followed for six months. The patient characteristics are presented in Table 1.

**Table 1:** Patient characteristics

**Analysis**	**All (n = 121)**	**Responders (n = 73)**	**Non-responders (n = 48)**	***P*-value**
Age, mean (years)	60.5 ± 14.0	58.2 ± 14.8	64.0 ± 11.9	0.023
Sex (M/F)	31/90	19/54	12/36	0.899
Current smoking	19/121 (16%)	11/73 (15%)	8/48 (17%)	0.813
RA duration, median (years)	10 (0 to 60)	11 (0 to 60)	8 (0 to 40)	0.60
Weight, mean (kg)	73.1 ± 16.4	72.1 ± 16.2	74.8 ± 16.8	0.375
HAQ index, mean	1.07 ± 0.64	1.08 ± 0.67	1.05 ± 0.60	0.791
DAS28 index, mean	4.40 ± 1.20	4.53 ± 1.22	4.21 ± 1.52	0.145
CRP, median (mg/L)	10.8 (0.1 to 159)	12.9 (0.2 to 159)	9.4 (0.7 to 119)	0.14
Larsen-Dale index, mean	1.50 ± 1.3	1.2 ± 1.06	1.9 ± 1.45	**0.002**
Synovial fluid, median (ml)	10 (0 to 105)	8 (0 to 105)	16.5 (0 to 75)	0.006

Mean ± SD, Median (range), Bold figures: *P* <0.003. CRP, C-reactive protein; DAS28, disease activity score in 28 joints; F, female; HAQ, Health Assessment Questionnaire; M, male; n, number; RA, rheumatoid arthritis; SD, standard deviation.

Most patients (89%) were using disease modifying antirheumatic drugs (DMARD). In all, 74 patients were treated with methotrexate alone or in combination with other DMARD, and biological agents were used by 24 participants. There was no significant difference in pharmacotherapy between the groups at baseline. During the observation period 22% of responders and 14% of non-responders changed their DMARD therapy (*P* = 0.189).

Oral glucocorticoids were used by 44% of the patients. There was no significant difference in treatment outcome between the subgroups with and without oral glucocorticoids (*P* = 0.265). Furthermore, there was no significant difference in number of additional IAGC treatments given for other joints (24 and 16 joints, respectively) between responders and non-responders during the observation period (*P* = 0.374).

Thirty-nine patients had never had a knee joint injection before and their responses to IAGC were similar to other patients (data not shown). A total of 73 of the 121 patients (60%) had no relapse during the six-month observation period (responders). The shortest time to relapse in the study was five days. In eight cases recurrence of knee symptoms was not associated with clinical synovitis. Mean duration to relapse in the non responding group was 88 ± 49 days and the participants in this group were older and had significantly more joint destruction in the knee (Table 1). Age and disease duration correlated to radiographic destruction (rho: 0.277, *P*: 0.003 and rho: 0.342, *P*: <0.001, respectively). The results of the laboratory analyses of serum samples are presented in Table 2. No significant differences between responders and non-responders for the analysed serum variables were found.

**Table 2:** Serum analysis

**Analysis**	**All (n = 121)**	**Responders (n = 73)**	**Non-responders (n = 48)**	***P*-value**
s-TNF (pg/mL)	0 (0 to 1,212)	0 (0 to 1,212)	0 (0 to 99)	0.399
s-IL6 (pg/mL)	14 (0 to 324)	30 (0 to 24)	7 (0 to 324)	0.629
s-VEGF (pg/mL)	218 (0 to 1,750)	218 (0 to 1,188)	212 (0 to 1,750)	0.850
s-MMP3 (pg/mL)	138 (16 to 116,714)	138 (16 to 116,714)	140 (34 to 52,031)	0.911
s-COMP (U/L)	10.7 ± 3.4	10.8 ± 3.9	10.5 ± 2.5	0.636
s–anti-CCP (>7 IU/ml)	87/121 (72%)	48/73 (66%)	39/48 (81%)	0.064
s-IgM-RF (>5 IU/ml)	90/121 (74%)	51/73 (70%)	39/48 (81%)	0.160
s-ANA	38/99 (38%)	14/39 (36%)	24/60 (33%)	0.682

Median (range), mean ± SD. ANA, antinuclear antibodies; anti-CCP, anti-cyclic citrullinated peptide; COMP, cartilage oligomeric matrix protein; IgM, immunoglobulin M; MMP3, matrix metalloproteinase 3; RF, rheumatoid factor; s, serum; VEGF, vascular endothelial growth factor.

**Figure 1:**
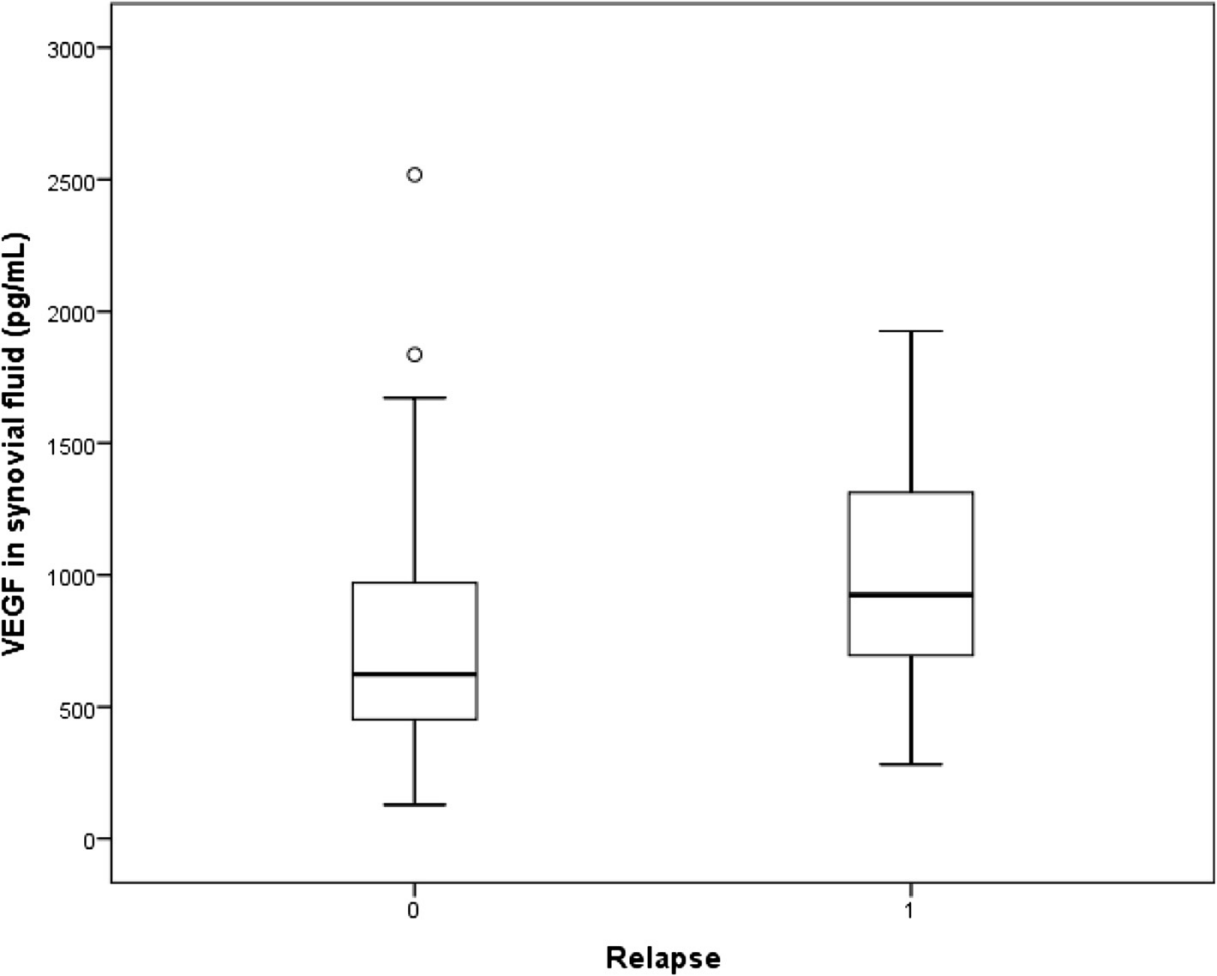
**Synovial fluid levels of VEGF among responders and non reponders.** VEGF, vascular endothelial growth factor.

Synovial fluid was available from 105/121 knees (87%) with no significant difference between groups. However, the effusion volumes were significantly larger for non-responders than for responders (Table 1). The level of VEGF in synovial fluid was significantly higher in non-responding patients (Table 3, Figure 1) and correlated significantly with IL6 levels in the synovial fluids (rho: 0.386; *P*: <0.001). No other differences between the groups were found. The results of the laboratory analysis of synovial fluid samples are presented in Table 3. After logistic regression analysis the findings for the Larsen-Dale score and VEGF in synovial fluid in relation to response remained.

**Table 3:** Synovial fluid analysis

**Analysis**	**All (n = 105)**	**Responders (n = 61)**	**Non-responders (n = 44)**	***P*-value**
sf-TNF (pg/mL)	42 (0 to 1,128)	51 (0 to 1,128)	36 (0 to 182)	0.706
sf-IL6 (pg/mL)	734 (0 to 10,000)	636 (0 to 10,000)	1,062 (0 to 10,000)	0.625
sf-VEGF (pg/mL)	778 (128 to 2,518)	622 (128 to 2.518)	998 (282 to 1,926)	**0.002**
sf-MMP3 (pg/mL)	5,367 (64 to 143,896)	6,696 (64 to 143,896)	5189 (524 to 72,922)	0.686
sf-COMP (U/L)	58.5 ± 36.0	54.4 ± 27.1	63.6 ± 44.6	0.220
sf-Mono (x10^9^/L)	1.7 (0 to 132.6)	1.9 (0 to 132.6)	1.5 (0 to 7.1)	0.208
sf-Poly (x10^9^/L)	1.65 (0 to 32.1)	1.6 (0 to 19.5)	1.7 (0 to 32.1)	0.801

Median (range), mean ± SD, Bold figures: *P* <0.003. COMP, cartilage oligomeric matrix protein; MMP3, matrix metalloproteinase 3; SD, standard deviation; sf, synovial fluid; VEGF, vascular endothelial growth factor.

Serum levels of TNF were only detectable in 35% and IL6 in 50% of the cases. Compared with serum, the synovial fluid levels of TNF, IL6, MMP3, COMP and VEGF were all significantly higher (*P* <0.001) (data not shown).

## Discussion

The main finding in the present study is that non-responders had more joint destruction and higher levels of VEGF in synovial fluid than responders. Neither the autoimmune profile, the ongoing cartilage turnover nor local or systemic inflammation seems to influence the relapse rate.

In this large prospective cohort study we have analysed several possible outcome predictors of IAGC for knee synovitis. Many previous outcome investigations include patients with different diagnoses. As different joints and different rheumatic diseases may have different predictors, in this study we have focused on knee injections in RA patients.

Smoking has been identified as an exogenous factor of importance in the pathogenesis of RA. Furthermore, treatment with methotrexate and biological agents is less successful in smokers [14], but results in the present study indicate that smoking has no influence on the outcome of IAGC in RA.

Postinjection rest of weight-bearing joints has been shown to improve the response to IAGC in arthritis (4), but our findings show that the body weight itself did not differ between responders and non-responders.

In a randomised controlled trial Jahangier *et al*. [7] compared knee injections with the combination of yttrium-90 + 20 mg THA with the combination of placebo + 20 mg THA on 66 patients with different arthritides. The overall response rate was 48% and no significant difference between the groups was found at six months. Pretreatment synovial biopsies showed significantly more synovial macrophages in patients responding on IAGC. Furthermore, Luukiainen *et al*. [6] studied 30 RA patients with knee synovitis after IAGC with 30 mg THA and found a significant correlation between polymorphonuclear leucocytes in synovial fluid and reduction of joint circumference after six months. This parameter had no influence on the relapse rate in our study.

The CIMESTRA study [8] was designed as a randomised controlled trial on recent onset RA with intense IAGC (14 mg betamethasone for each knee injection) combined with either cyclosporine A and methotrexate or sulfasalazine and methotrexate. The tight control regimen used made early detection of arthritis relapse possible. A 48% relapse rate after 200 days was found among the 89 included patients with knee synovitis. In the present investigation we used 20 mg THA on patients with longer disease duration (median 10 years) and found a 40% relapse rate after 180 days and we defined relapse as a request for another injection confirmed with synovitis on clinical examination. Despite differences in study design and relapse definition the difference in general relapse rate between the studies is small.

The CIMESTRA trial showed that the effect of the first joint injection had a longer duration than a subsequent injection in the same joint and the presence of anti-CCP was weakly associated with longer injection survival. This is not confirmed in our study, which, in contrast, showed a non-significant trend towards more relapse among the anti-CCP positive patients.

In a study by af Klint *et al.*, 31 patients were examined with synovial biopsies before and two weeks after IAGC using 40 mg THA for knee synovitis [15]. The results showed marked reduction of synovial T-lymphocytes, TNF, IL1 and VEGF, but no significant change in synovial vascularity or the expression of VEGF on endothelial cells. The authors discussed whether this finding was important for the transient effect of IAGC. This hypothesis is supported by our finding of significantly higher levels of VEGF in synovial fluid of non-responders in the present study.

VEGF is normally produced by synovial cells in response to hypoxia and proinflammatory cytokines, such as TNF and IL6 [16]. In accordance, levels of IL6 and VEGF in synovial fluid correlate strongly in our study. VEGF stimulates the formation of new blood vessels and increases vascular permeability. This is important for production of the synovial fluid that represents an ultrafiltrate of plasma as the synovial membrane lacks basement membranes towards the synovial cavity. VEGF may also protect synoviocytes from apoptosis [17], thereby further contributing to synovial hyperplasia. The high levels of VEGF in the synovial fluid of IAGC non-responders in the present study may have stimulated synovial vascularity and rapid recurrence of blood perfusion and probably the larger joint effusion as well. This facilitates recruitment of inflammatory cells and may explain the increased risk for relapse. The fact that this difference in VEGF levels between responders and non-responders was found locally in the treatment target organ, but not systemically, further argues for a pathogenetic impact of VEGF in the joints in steroid-resistant RA patients. The role of sf-VEGF in this study is in agreement with animal studies on the role of VEGF in arthritis. VEGF knockout mice showed reduced pathology and less synovial angiogenesis in both antigen-induced and collagen-induced models of arthritis [18]. An anti-VEGF antibody has also been shown to prevent collagen-induced arthritis and reduced established disease activity in mice [19]. Antibodies to VEGF receptor 1 have also been shown to attenuate disease activity in mice [20]. These findings suggest the possibility that local anti-VEGF administration might be an adjunct therapy to THA in RA patients with high synovial fluid levels of VEGF.

Compared to synovial fluid, the VEGF levels in serum are significantly lower and show no difference between the responder groups, indicating that VEGF levels or VEGF-associated pathology in the local knee environment determines treatment outcome. None of the systemic variables analysed seems to be important, suggesting a limited value of analysing serum samples before IAGC to predict clinical outcome.

However, in juvenile chronic arthritis a high erythrocyte sedimentation rate was correlated with a better response to IAGC for knee synovitis [21]. Remission of synovitis of the knee after IAGC lasted longer, not only if concomitantly treated with methotrexate, but also with a procedure including general anaesthesia [22]. The latter finding was unexpected and the authors discussed whether the injection placement in a child is easier during general anaesthesia.

Not surprisingly, the radiographic changes in our patients were correlated both with disease duration and age. For elderly RA patients comorbidity with osteoarthritis (OA) cannot be excluded. How these joint diseases interact is not known, and comparing the outcome of IAGC between OA and RA is difficult because of differences in pathogenesis, but in the late stages of both diseases there are more mechanical than immunological causes for inflammation. RA patients with severe joint destruction may, therefore, respond more like patients with severe OA. A systematic review of outcome predictors in OA [23] concluded that results are not consistent, but some of the reviewed studies suggest that the presence of effusion and the severity of disease have an impact on treatment outcome. As in several OA studies, we found an association between degree of joint damage on radiographs and outcome of IAGC. This has never been shown in RA before. COMP levels, however, did not differ between responders and non-responders, which suggest that the ongoing cartilage turnover has only minor effects on the effect of IAGC in RA.

A weakness of the study may be the relapse definition, based on the complaints of the patient, and a confirming clinical examination. The patient delay may vary due to individual thresholds for discomfort, pain and need for help. On the other hand, this method has been used before [3,24] and is relevant because it reflects the real life in clinical practice.

In this investigation we have chosen to use 20 mg THA, as this is the recommended dose for knee injections in Sweden. Higher doses are often used, but there are no published dose finding studies comparing the efficacy of different THA dosages for knee injections, and consequently, there is a large variation in the tradition of using THA.

## Conclusions

The present study suggests that synovial fluid levels of VEGF have an impact on treatment outcome. In addition to radiological joint damage, it is the best predictor of IAGC outcome for knee synovitis and this finding deserves further research. A clinical application of our findings is that RA patients with severe radiographic joint changes should benefit from extra careful injection procedures, complete synovial fluid aspirations, strict postinjection immobilisation and tighter follow up. Ultrasound-guidance may also improve the injection outcome, but results are conflicting [25,26].

In patients with relapsing synovitis despite normal radiographs and accurate IAGC, a joint infection should be excluded. If cultures are negative, analysis of VEGF in synovial fluid might be helpful. Our results suggest that intra-articular anti-VEGF therapy in the future might be considered as an adjunct therapy in RA patients with relapsing synovitis and high VEGF levels in synovial fluid.

## Abbreviations

ANA: antinuclear antibodies; CCP: cyclic citrullinated peptide; COMP: cartilage oligomeric matrix protein; CRP: C-reactive peptide; DAS: disease activity score; DMARD: disease modifying antirheumatic drugs; ELISA: enzyme-linked immunoabsorbent assay; HAQ: Health Assessment Questionnaire; IAGC: intra-articular glucocorticoid treatment; IL: interleukin; MMP: matrix metalloproteinase; MRI: magnetic resonance imaging; OA: osteoarthritis; RA: rheumatoid arthritis; RF: rheumatoid factor; SD: standard deviation; SF: synovial fluid; THA: triamcinolone hexacetonide; TNF: tumour necrosis factor; VEGF: vascular endothelial growth factor.

## Competing interests

TS is cofounder and owns stocks in AnaMar. The other authors declare that they have no competing interests.

## Authors’ contributions

All co-authors fulfilled the criteria for authorship. TW initiated and designed the study. TW, AK and JL participated in the management of the patients and their recruitment in the cohort and in the acquisition of data. JR contributed with laboratory analysis of autoantibodies. TS performed the COMP analysis and AL the laboratory analysis of all inflammation markers. TW, JR, AK, JL, TS and AL all contributed with interpretation of data. TW drafted the manuscript and JR, AK, JL, TS and AL critically revised the manuscript. All authors have read and approved the final manuscript and agree to be accountable for all aspects of the work.
